# Case Report: Zero-fluoroscopy retrograde transcatheter closure of a ruptured Sinus of valsalva guided by transesophageal echocardiography

**DOI:** 10.3389/fcvm.2026.1906135

**Published:** 2026-09-14

**Authors:** Damba Dwisepto Aulia Sakti, Yovi Kurniawati, Rina Ariani, Leroy Leon Leopold Lasanudin, Abraham Tombe, Radityo Prakoso

**Affiliations:** 1 Department of Cardiology and Vascular Medicine, Faculty of Medicine, Universitas Indonesia, National Cardiovascular Center Harapan Kita, Jakarta, Indonesia; 2 Faculty of Medicine, Universitas Indonesia, Jakarta, Indonesia

**Keywords:** case report, ruptured sinus of valsalva, transcatheter closure, transesophageal echocardiography, zero-fluoroscopy

## Abstract

**Background:**

Ruptured sinus of Valsalva (RSOV) is a rare cardiac lesion that may lead to a progressive right-sided cardiac overload and ultimately heart failure. Although surgery remains the primary intervention, transcatheter closure has emerged as a less invasive alternative in suitable patients. However, zero-fluoroscopy retrograde transcatheter closure of RSOV under echocardiography guidance alone is extremely rare.

**Case presentation:**

A 31-year-old man with a history of hypertension presented with symptoms of atypical chest pain and exertional dyspnea. Heart auscultation showed a grade III/VI with a to-and-fro murmur. Pre-procedural transthoracic and transesophageal echocardiography (TTE and TEE) demonstrated a ruptured aneurysm from the right coronary cusp of the sinus of Valsalva with shunting into the right atrium. The defect measured 7 mm at the aortic end and 6 mm at the right atrial end, with a distance of 16.5 mm from the right coronary ostium and no significant aortic regurgitation. The patient underwent a zero-fluoroscopy percutaneous closure using a retrograde transarterial approach under echocardiography guidance only. A Konar-MF VSD Occluder (8/10 mm) was successfully deployed. Post-procedural imaging showed a stable device position, with mild central residual shunt. No obstruction of the right coronary ostium or aortic regurgitation was observed. Final follow-up on TTE examination at 2 years revealed a complete closure without any residual flow.

**Conclusion:**

This case demonstrates the technical feasibility of a fully TEE-guided, zero fluoroscopy, retrograde transcatheter closure of RSOV in patients with favorable anatomy. This approach reduces radiation and contrast exposure without altering the short-term procedural success.

## 1 Introduction

Sinus of Valsalva aneurysm (SOVA) is an uncommon pathological condition arising from weakness in the elastic lamina of the aortic wall, with a reported general incidence of 0.09% (1). SOVA most commonly originates from the right coronary sinus (approximately 70%–75%), followed by the non-coronary sinus, with left coronary sinus involvement being rare (2). When the aneurysmal wall perforates into an adjacent cardiac chamber, a ruptured sinus of Valsalva (RSOV) develops, creating a left-to-right shunt. Without intervention, RSOV leads to progressive volume overload of the right heart and lungs, and ultimately heart failure.

Historically, and still today, surgery remains the gold standard for RSOV repair. This allows a direct view of the windsock and simultaneous correction in cases with accompanying defects, such as ventricular septal defect (VSD) or aortic regurgitation (AR). Large series and reviews report operative mortality rates of up to 4%, with postoperative lengths of stay ranging from 7.2 to 10.3 days. Long-term surgical outcomes estimate a 10-year survival rate of over 90%, and low recurrence when patch closure is used (3–5). In recent years, transcatheter closure has emerged as a less invasive alternative, demonstrating a high procedural success rate, low mortality, and shorter hospital stay compared with surgical repair (6, 7).

Despite this progress, transcatheter procedures rely heavily on fluoroscopic guidance, often in combination with transesophageal echocardiography (TEE) or angiography. The use of fluoroscopy and contrast media is not without risk. Contrast-associated acute kidney injury (CA-AKI) has been reported in 1%–2% of the general cardiac catheterization population, rising substantially in patients with pre-existing chronic kidney disease or diabetes (8). In children undergoing cardiac catheterization, the estimated lifetime risk of fatal radiation-induced malignancy is 0.07%–0.08% (9). These risks have catalyzed a growing interest in a radiation-free approach for cardiac procedures.

Zero-fluoroscopy catheter-based procedures have been reported in electrophysiological ablations and structural interventions, such as atrial septal defect (ASD) closures (10, 11). In contrast, zero-fluoroscopy transcatheter closure of RSOV has not been systematically studied, and published cases are isolated. To our knowledge, the present case is the first to document a fully TEE-guided, zero-fluoroscopy, retrograde transcatheter RSOV closure. We report the procedural technique, outcomes, and discuss patient selection criteria, device choice, as well as the role of TEE in replacing fluoroscopic guidance for this specific lesion.

## 2 Case description

### 2.1 History and examination

A 31-year-old male patient with a known history of hypertension presented to our center with atypical chest pain and progressively worsening exertional dyspnea. Physical examination revealed a blood pressure of 140/78 mmHg, heart rate of 96 beats per minute, and oxygen saturation of 96% on room air. A grade III/VI to-and-fro murmur was detected from cardiac auscultation. The patient was examined using a 12-lead electrocardiogram, showing a normal sinus rhythm with slight right axis deviation, which possibly reflects a right ventricular volume loading from the left-to-right shunt. Left ventricular hypertrophy exists without any ischemic signs or conduction abnormalities. Chest radiography also revealed cardiomegaly.

Bedside transthoracic echocardiography (TTE) and TEE confirmed a ruptured aneurysm in the right coronary cusp of the sinus of Valsalva with shunting into the right atrium (Figure 1). The lesion was classified as type IIIa according to the classic Sakakibara-Konno classification, as the aneurysm originated from the right coronary sinus and ruptured into the right atrium. Furthermore, it corresponded to type II Guo classification, indicating that the right atrial rupture site was adjacent to the tricuspid annulus (12, 13). The defect measured 7 mm at the aortic end and 6 mm at the right atrial end, with a distance of 16.5 mm between the defect and the right coronary ostium. No associated VSD, aortic regurgitation, or other structural abnormality was identified.

**Figure 1:**
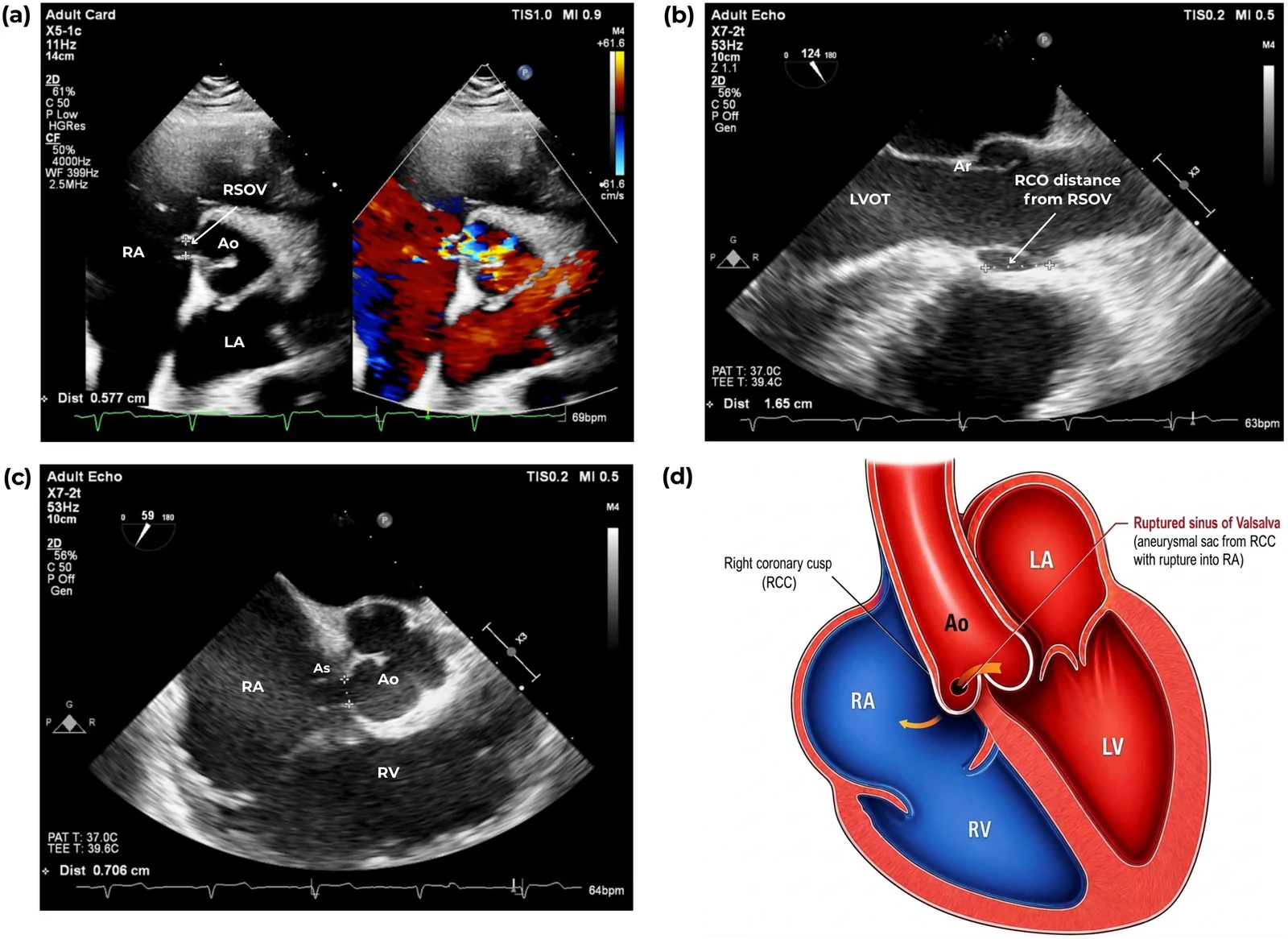
Baseline TTE and TEE assessment of RSOV. **(a)** Parasternal short-axis TTE grayscale and color Doppler images showed a ruptured aneurysm arising from the RCC of the sinus of Valsalva and protruding into the RA, with continuous turbulent left-to-right shunting from the Ao into the RA; the RA end of the defect measured approximately 6 mm. **(b)** Clockwise rotation of the LVOT view TEE image at 124° demonstrated the distance between the rupture tract and the RCO, measuring approximately 16.5 mm, the LVOT is also shown. **(c)** The TEE image at 59° demonstrated the aortic end of the defect, measuring approximately 7 mm. No AR or MR was identified. TR could not be adequately assessed because of interference from the RSOV jet. **(d)** Schematic illustration of the baseline anatomy showing RSOV arising from the RCC with rupture into the RA, resulting in a left-to-right shunt from the Ao to the RA. Ao, aorta; AR, aortic regurgitation; Ar, aortic root; As, aneurysmal sac; LA, left atrium; LV, left ventricle; LVOT, left ventricular outflow tract; MR, mitral regurgitation; RA, right atrium; RCC, right coronary cusp; RCO, right coronary ostium; RSOV, ruptured sinus of Valsalva; RV, right ventricle; TEE, transesophageal echocardiography; TR, tricuspid regurgitation; TTE, transthoracic echocardiography.

Echocardiography also showed a dilated right atrium and right ventricle, consistent with right-sided volume overload secondary to a continuous left-to-right shunt. Right ventricular systolic function remained preserved, with tricuspid annular plane systolic excursion (TAPSE) of 26 mm. Left ventricular systolic function was also preserved with an LVEF of 63%. This suggests that the left ventricular hypertrophy is linked to the patient’s hypertension, while the accompanying right atrial and right ventricular dilatation are caused by shunting of the heart, which has hemodynamic significance.

Color Doppler confirmed a continuous left-to-right flow from the aorta into the right atrium. Tricuspid regurgitation could not be adequately assessed due to the interference from the RSOV jet. Therefore, pulmonary artery systolic pressure could not reliably estimate the tricuspid regurgitation velocity. The pulmonary artery acceleration time was shortened to 69 ms, with an acceleration slope of 1,524 cm/s², in an estimated mean pulmonary artery pressure of 48 mmHg. The pulmonary-to-systemic blood flow ratio (Qp/Qs) was not measured because right heart catheterization with an oximetry run was intentionally omitted as part of the planned zero-fluoroscopy strategy. Nevertheless, transcatheter closure was deemed appropriate based on the presence of a continuous left-to-right shunt, progressive right-sided chamber dilatation, and worsening exertional dyspnea.

### 2.2 Diagnostic assessment

Differential diagnosis of a near-continuous murmur and left-to-right shunting was also considered (14, 15). Patent ductus arteriosus (PDA) was excluded based on the TTE and TEE results, which demonstrated no communication between the aorta and pulmonary artery. A coronary-cameral fistula was also unlikely because the shunt originated from an aneurysmal right coronary sinus rather than a coronary artery, with no evidence of coronary artery dilatation or an independent fistulous tract. VSD was excluded by the presence of an intact interventricular septum and the absence of an associated interventricular communication. In addition, normal aortic valve morphology without evidence of aortic stenosis or regurgitation, excluded valvular pathology as the cause of the murmur. Collectively, the echocardiographic findings demonstrating a direct communication between the right coronary sinus and the right atrium established the diagnosis of RSOV (14, 16).

### 2.3 Procedural planning and decision-making

Following a multidisciplinary cardiac team discussion, we decided to pursue percutaneous transcatheter closure. The rationale for a minimally invasive approach primarily relies on the isolated, well-circumscribed RSOV with favorable dimensions and adequate distance from the right coronary ostium. There was also no significant aortic regurgitation or associated defects requiring surgical correction. The optimal echocardiographic image quality provides all the anatomical information required for device selection and guidance. TEE imaging provided superior real-time delineation of the RSOV anatomy and its relationship to the aortic valve and coronary ostia compared to aortography alone. Thus, elimination of fluoroscopy was considered technically feasible for this patient.

### 2.4 Intervention

The procedure was performed under general anesthesia with continuous TEE monitoring. Fluoroscopy was available as a backup but was not used, and no iodinated contrast was given. Prophylactic antibiotics using 2,000 mg of intravenous cefazolin were administered prior to vascular access. The right femoral artery was then punctured, and a 6-curve, 7F guide slender sheath was inserted. Unfractionated heparin 5,000 IU was administered through the arterial sheath. Activated clotting time (ACT) monitoring was not performed during the procedure.

Under real-time TTE and TEE guidance, a JR Guiding catheter 3.5 curve, 7F guide assisted by a 0.035-inch soft hydrophilic Terumo guidewire was advanced retrogradely through the descending aorta, aortic arch, and aortic root (Figure 2). The procedural images documented clockwise rotation of the left ventricular outflow tract (LVOT) view, predominantly at multiplane angles of 91–107°, which allowed visualization of the guidewire, catheter tip, RSOV tract, and adjacent right atrium (Figure 2). The imaging plane was adjusted continuously, with close communication between the interventional echocardiographer and the operator. Advancement was paused whenever the wire or catheter tip could not be clearly identified and resumed only after its position had been re-established.

**Figure 2:**
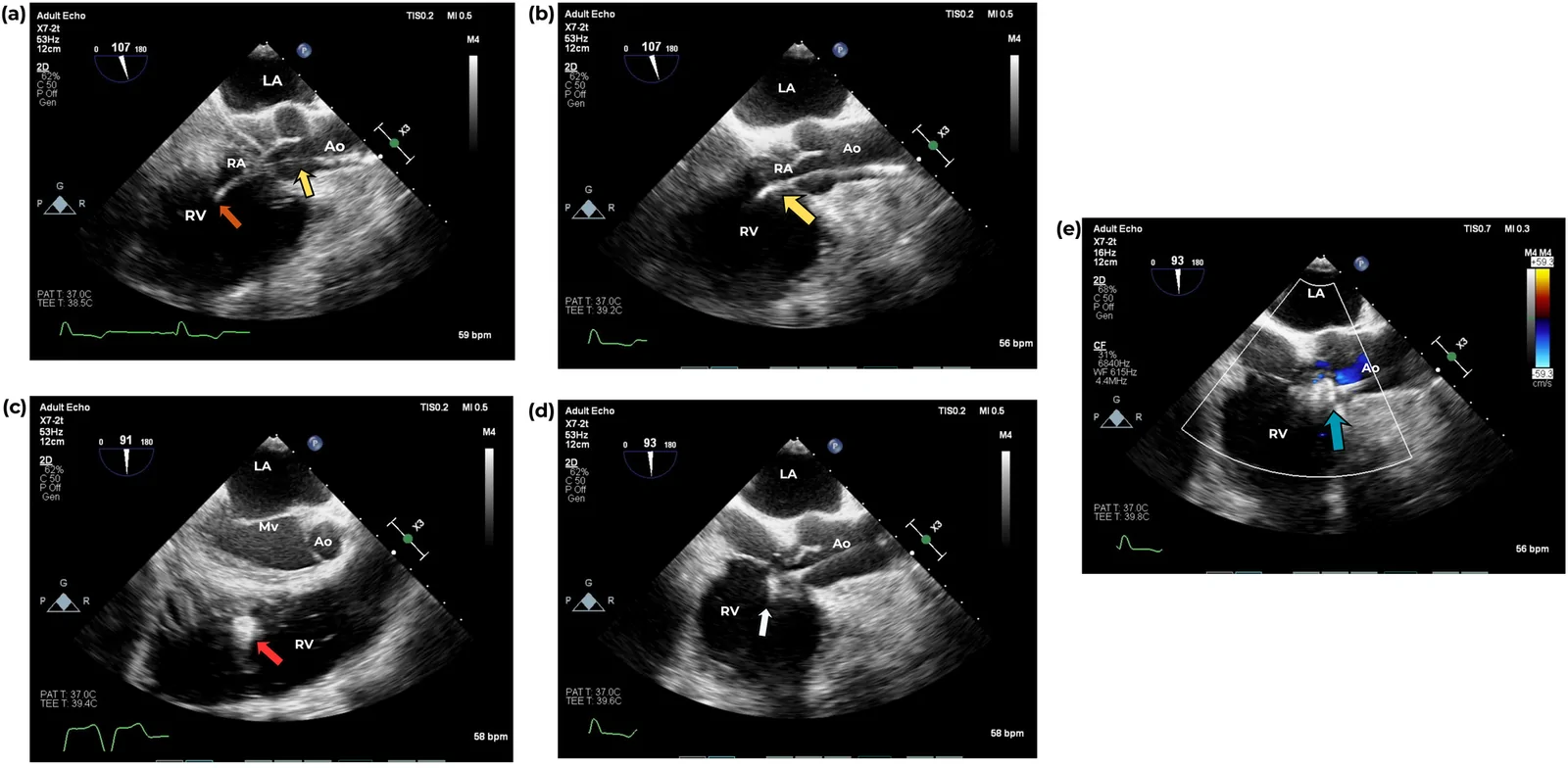
Step-by-step intra-procedural TEE guidance of zero-fluoroscopy retrograde transcatheter RSOV closure. All images were obtained using an LVOT view with clockwise rotation of the TEE probe. **(a)** A 0.035-inch soft hydrophilic guidewire was advanced through the 7-Fr JR 3.5 guiding catheter, across the RSOV and right atrium, and into the right ventricle. The distal guidewire within the right ventricle and the guiding catheter across the defect are indicated by the orange and yellow arrows, respectively. **(b)** The guiding catheter was advanced further over the guidewire into the mid-right ventricle while the guidewire was gradually withdrawn. (Yellow Arrow) **(c)** The Konar-MF ventricular septal defect occluder No. 8/10 mm was advanced through the catheter until it reached the tip of the delivery system. **(d)** The low-pressure disc was deployed at the right ventricular side, and the entire system was retracted into the aneurysmal sac. **(e)** High-pressure disc deployed at the aortic end of the defect with color Doppler confirming reduced shunting. Ao, aorta; LA, left atrium; MV, mitral valve; RA, right atrium; RSOV, ruptured sinus of Valsalva; RV, right ventricle; TEE, transesophageal echocardiography.

The catheter is carefully navigated, starting from the RSOV into the right atrium, then into the right ventricle. Finally, the guiding catheter was advanced over the wire into the defect and placed in the middle of the right ventricle. A Konar-MF VSD Occluder No. 8/10 mm was delivered through the catheter and advanced to the catheter tip. The low-pressure disc was deployed at the right ventricle. The entire system was gently withdrawn towards the aorta until the low-pressure disc was opposed to the right atrial exit of the RSOV. The high-pressure disc was subsequently deployed at the aortic end within the right coronary sinus.

Before the final release, the device was assessed for stability using a gentle push-pull maneuver under real-time TEE guidance. This confirmed the device in satisfactory alignment, with the high-pressure disc positioned on the aortic side and the low-pressure disc on the right atrial side of the ruptured tract. Color Doppler examination revealed a mild residual shunt without any new aortic regurgitation, and no signs of blocking in the right coronary ostium. The arterial sheath was then removed following the device release. Hemostasis was successfully achieved by gentle manual compression for one hour. The total procedural duration was 43 min, with an estimated blood loss of approximately 15 mL. TEE guidance can be properly maintained throughout the procedure.

### 2.5 Outcomes

After the device was released, an LVOT view with clockwise rotation was obtained, complemented by a TTE parasternal short-axis and apical four-chamber view. It showed no right coronary ostial obstruction, aortic regurgitation, or pericardial effusion. The RSOV jets were reduced, with a mild tricuspid regurgitation (Figure 3). The patient was monitored overnight and discharged the following day in a stable condition. No additional bleeding or complications were observed during the post-procedural period. At discharge, the patient was prescribed 80 mg of aspirin once daily.

**Figure 3:**
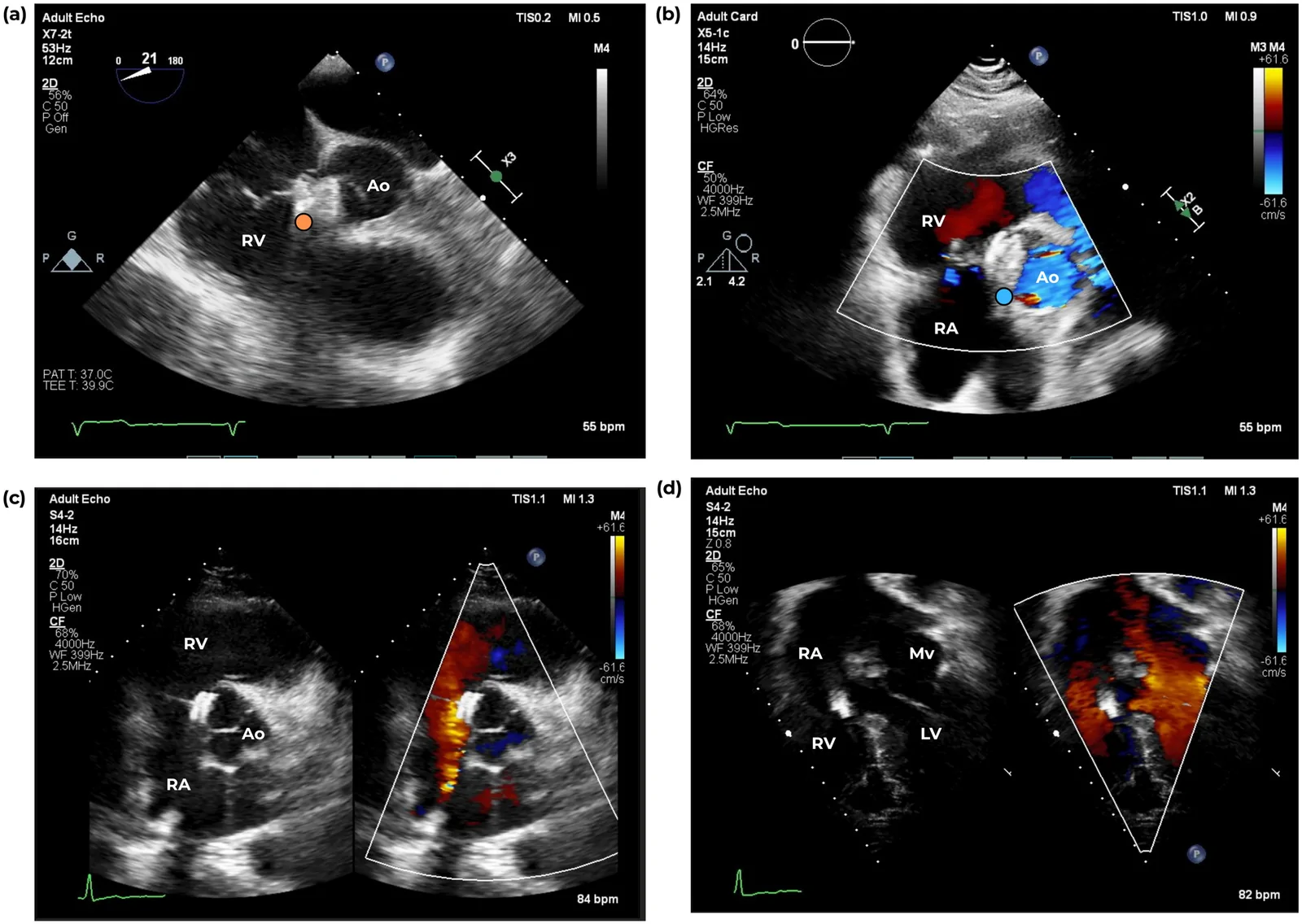
Immediate and long-term post-procedural echocardiographic assessments. **(a)** The TEE short-axial grayscale image demonstrated good final device position (Orange Circle) across the RSOV after occluder release. **(b)** Post-procedural parasternal short-axis TTE color Doppler image demonstrated mild central residual shunt across the device (blue circle). RCA ostium obstruction was not found, and no AR was found, while mild TR was present **(c,d)**. Two-year follow-up parasternal short-axis and apical four-chamber TTE images with color Doppler showed a stable occluder device position and no significant residual shunt, supporting durable closure after the procedure. Ao, aorta; AR, aortic regurgitation; LA, left atrium; LV, left ventricle; LVOT, left ventricular outflow tract; MV, mitral valve; RA, right atrium; RSOV, ruptured sinus of Valsalva; RV, right ventricle; TEE, transesophageal echocardiography; TR, tricuspid regurgitation; TTE, transthoracic echocardiography.

Post-procedure ECG revealed a normal sinus rhythm without any new ischemic changes. TTE performed on day 1 and at one-week post-procedure demonstrated a stable device position without evidence of embolization, pericardial effusion, or new valvular pathology. At the 10-month follow-up, TTE confirmed complete closure of the defect with no residual flow. Long-term follow-up TTE at 2 years demonstrated a sustained device stability, the absence of significant residual shunt, and no aortic regurgitation or device-related complications (Figure 4). The patient remained clinically stable.

**Figure 4:**
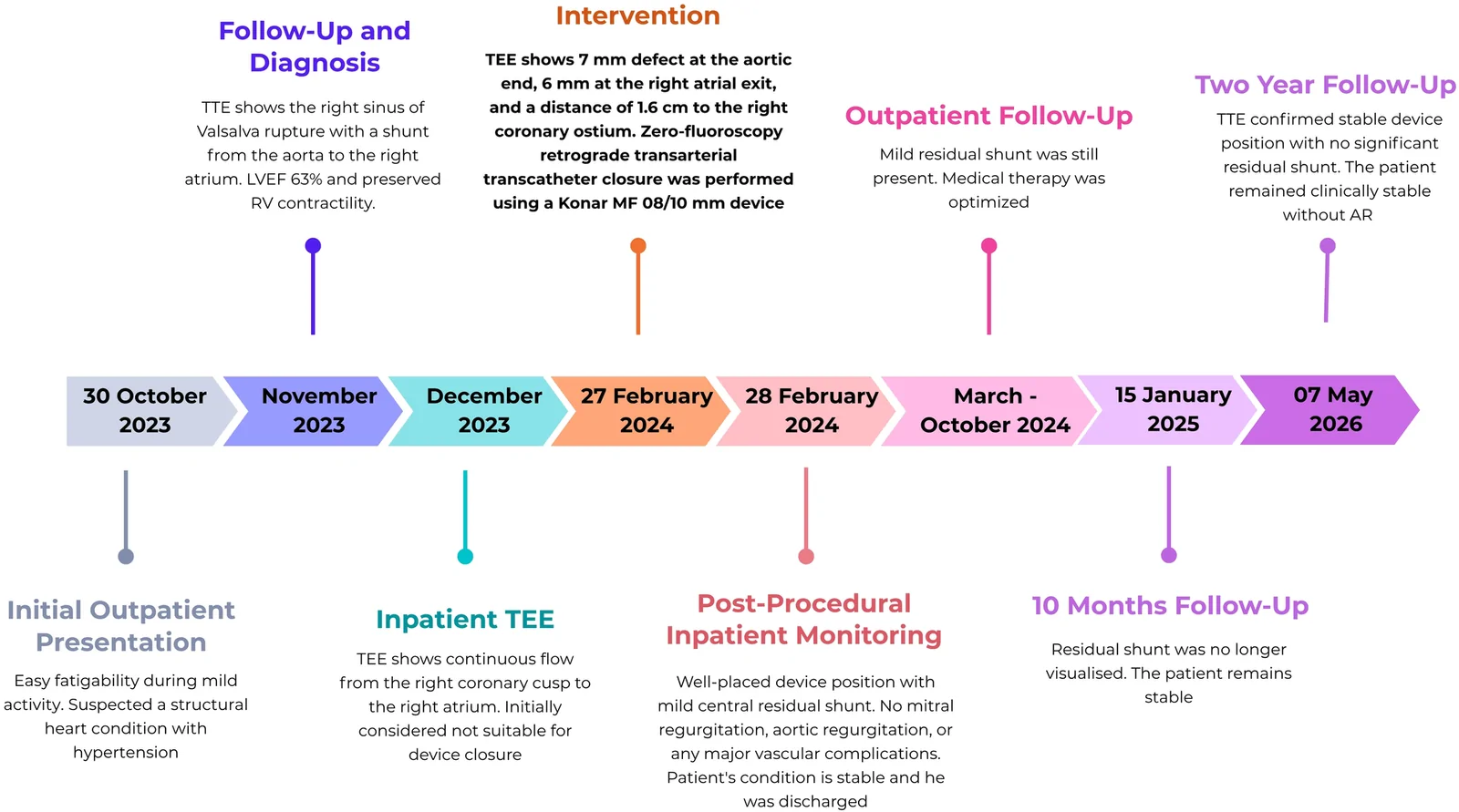
Timeline of the patient’s clinical course, including initial outpatient presentation, diagnostic echocardiographic evaluation, inpatient TEE, zero-fluoroscopy retrograde transcatheter closure of RSOV, post-procedural monitoring, and follow-up outcomes. LVEF, left ventricular ejection fraction; RA, right atrium; RCC, right coronary cusp; RV, right ventricle; RSOV, ruptured sinus of Valsalva; TEE, transesophageal echocardiography; TTE, transthoracic echocardiography.

### 2.6 Patient perspective

Following a comprehensive discussion of the procedural strategy, including the rationale for a fluoroscopy-free approach, the patient consented to undergo the intervention under TEE guidance alone. The procedural techniques, potential benefits, risks, and alternative treatment options were thoroughly explained. After the intervention, the patient experienced marked symptomatic improvement without procedure-related complications and was satisfied with the outcome.

## 3 Discussion

Zero-fluoroscopy, especially echocardiography-guided transcatheter closure, has previously been described for ASD cases (10, 17). Retrograde transcatheter closure of RSOV with TEE support has also been reported; however, these procedures continued to rely on fluoroscopic guidance (18). To the best of our knowledge, this is the first reported case of a zero-fluoroscopy, TEE-guided, retrograde transcatheter closure of an RSOV. The novelty of this case, therefore, lies in the combination of these elements.

The case illustrates three clinically important advances in structural cardiology intervention: (1) the technical feasibility of eliminating fluoroscopy for RSOV closure in an anatomically suitable patient; (2) the ability of TEE to provide sufficient procedural guidance in carefully selected patients; and (3) the suitability of the Konar-MF VSD Occluder for retrograde deployment in this uncommon defect morphology.

RSOV typically manifests in young to middle-aged adults, with a male preponderance and higher prevalence in Asian populations (2). Thus, this case represents an adult presentation of a congenital heart disease. The presenting symptoms in the present case (progressive dyspnea and a to-and-fro murmur in a young hypertensive man) are characteristics of a hemodynamically significant left-to-right shunt and warrant prompt echocardiographic evaluation. The TTE and TEE findings, including the right coronary cusp origin, right atrial destination, windsock morphology, and preserved aortic valve function, defined an ideal candidate for percutaneous closure.

Surgery remains the standard modality when RSOV is complicated by associated defects requiring repair, such as VSD. It is also preferred when there is significant aortic regurgitation (AR), or when adverse congenital valve abnormalities are present. In isolated RSOV with favorable anatomy, transcatheter closure (TCC) has demonstrated comparable efficacy to surgery with a shorter hospital stay and lower procedural burden. A 2023 meta-analysis by Mao et al. confirmed that TCC reduces hospital length of stay by approximately 2 days, with no significant differences in mortality, residual shunting, or new aortic regurgitation when compared to surgery (6). A complementary systematic review by Ayati et al., including 407 TCC patients, reported an overall procedural success rate of 95.6% and a major complication rate of 12%, with only 1.7% sustaining significant residual shunts after discharge (7).

In our case, the justification for eliminating fluoroscopy rests on several reasons. First, the patient presented with an isolated defect that was well visualized on TTE and TEE. The defect dimensions were favorable for device implantation, with adequate distance from the right coronary ostium, and no significant aortic regurgitation. Second, iodinated contrast was avoided, eliminating the risk of CA-AKI, which in patients undergoing cardiac catheterization varies from 1%–2% in the general population to over 30% in high-risk subgroups (8). Lastly, radiation exposure was entirely averted. This is especially relevant given the patient’s young age and the cumulative risks associated with repeat catheterization and long-term follow-up imaging.

In contrast to traditional antegrade transcatheter RSOV closures, a retrograde transarterial technique was used. The standard antegrade technique involves creating an arteriovenous guidewire loop by crossing the RSOV from the inferior vena cava (IVC) into the right atrium after establishing transseptal access. This is substantially more complex to navigate under echocardiographic guidance alone, especially when there is a high-pressure gradient between the aorta and the right atrium. The retrograde approach, using a JR guiding catheter advanced through the aortic root directly into the RSOV, allows a more intuitive catheter trajectory that can be tracked on TEE without fluoroscopy. A 2025 case report by Bagur et al. further supports the retrograde technique as a simplified alternative that avoids the arteriovenous loop and its associated maneuvers (18).

Device selection is another critical determinant of procedural success and long-term durability. It is aimed to avoid disruption to surrounding structures, including the coronary ostium and aortic valve movement, thereby preventing aortic regurgitation or stenosis. To date, no device has received regulatory approval specifically for RSOV closure. PDA occluders have historically been the most frequently used devices, accounting for 41.7% of cases in the Ayati et al. systematic review, followed by the Amplatzer Ductal Occluder (20.6%) (7). The Konar-MF VSD Occluder was selected in the present case for its asymmetric dual-disc configuration, polytetrafluoroethylene (PTFE) membrane, and adaptable waist. This enabled stable anchoring across the RSOV windsock during controlled deployment while carefully preserving the aortic valve and the right coronary ostium without complications. An 8/10 mm Konar-MF device was selected based on the measured defect dimensions (7 mm aortic end), consistent with the convention of sizing devices 1–2 mm above the largest defect diameter (7).

An additional anatomical consideration is the relationship between the RSOV exit and the tricuspid valve. The distance between the RA defect and the tricuspid annulus of the present case was measured at 1 cm (Supplementary Figure S1). This is clinically relevant because the low-pressure disc may potentially interfere with tricuspid leaflet motion when the defect is in close proximity to the valve. In our case, TEE demonstrated stable device positioning without interference with tricuspid valve motion or development of significant tricuspid regurgitation.

Apart from the retrograde maneuver, device selection, and anatomical consideration, echocardiography played an indispensable role throughout the procedure. Pre-procedural TEE characterized the defect anatomy and defined the device size. A three-dimensional TEE further demonstrated the RSOV anatomy and its relationship with the surrounding cardiac structures (Supplementary Video S5). During the procedure, TEE acts as a substitute to fluoroscopy. It guided catheter navigation, confirmed disc deployment orientation, and provided real-time assessment of residual shunting and aortic valve function after device release. These capabilities mirror those described in the zero-fluoroscopy ASD closure literature, where TEE has been shown to provide sufficient spatial information for device guidance (10, 17). The mild residual shunt observed post-device deployment immediately is a recognized and usually transient phenomenon. Meanwhile, the complete closure confirmed at the 10-month follow-up is consistent with outcomes reported for similarly sized devices in the TCC literature.

Although the intervention was considered successful, several limitations should be acknowledged. First, this report describes a single case, limiting its generalizability to the broader population of RSOV patients. The feasibility of a completely fluoroscopy-free transcatheter approach may not extend to more complex anatomical or clinical scenarios, including defects located in close proximity to the coronary ostia, suboptimal echocardiographic visualization, hemodynamic instability, significant concomitant aortic regurgitation, or associated cardiac lesions requiring surgical intervention. Second, the success of a zero-fluoroscopy strategy is critically dependent on optimal TEE image quality and advanced interventional echocardiographer. This level of imaging quality, along with the interventionist expertise, may not be not found in all centers. Third, an invasive hemodynamic assessment, such as catheter oximetry of Qp/Qs, was not performed; the level of indication for shunt severity and closure was based solely on the patient’s clinical presentation and echocardiographic findings. Lastly, fluoroscopy should remain a necessary safety net in centers where echocardiographic visualization is suboptimal or catheter maneuvering is ambiguous, especially when there is any doubt regarding the wire position or the catheter. Fluoroscopy needs to be immediately available when the zero-fluoroscopy course is completed.

## 4 Conclusion

This case demonstrates the technical feasibility of a fully TEE-guided, zero-fluoroscopy, retrograde transcatheter closure of RSOV using a Konar-MF VSD Occluder in a carefully selected patient. The favorable procedural and 2-year follow-up outcomes further support the feasibility of this approach under certain anatomical conditions. In a patient with an isolated right coronary cusp RSOV, favorable defect dimensions, adequate coronary ostial clearance, preserved aortic valve function, and excellent echocardiographic windows, the complete elimination of fluoroscopic and contrast guidance did not compromise procedural precision or long-term outcomes. Prospective multi-center registries are needed to define formal patient selection criteria, standardize the zero-fluoroscopy TEE-guidance protocol for RSOV, and ensure long-term device durability data. Until such evidence is available, this approach should be considered in experienced centers with dedicated interventional echocardiography expertise, applied exclusively to patients whose anatomy satisfies the established criteria for safe percutaneous RSOV closure.

## Data Availability

The original contributions presented in the study are included in the article/Supplementary Material, further inquiries can be directed to the corresponding author.
